# Proximal aortic stiffening in Turner patients may be present before dilation can be detected: a segmental functional MRI study

**DOI:** 10.1186/s12968-017-0331-0

**Published:** 2017-02-13

**Authors:** Daniel G. H. Devos, Katya De Groote, Danilo Babin, Laurent Demulier, Yves Taeymans, Jos J. Westenberg, Luc Van Bortel, Patrick Segers, Eric Achten, Jean De Schepper, Ernst Rietzschel

**Affiliations:** 1 Department of Radiology, MRI (-1K12), Ghent University Hospital, De Pintelaan 185, B-9000 Gent, Belgium; 2 Pediatric Cardiology, Department of Pediatrics and Turner Clinic, Ghent University Hospital, De Pintelaan 185, B-9000 Gent, Belgium; 3 Telecommunications and Information Processing, TELIN-IPI-iMinds, Faculty of Engineering and Architecture, Ghent University, Sint-Pietersnieuwstraat 41, 9000 Ghent, Belgium; 4 Department of Cardiology, Ghent University Hospital, De Pintelaan 185, B-9000 Gent, Belgium; 5 Department of Radiology, Leiden University Medical Center, Albinusdreef 2, 2333 ZA Leiden, The Netherlands; 6 Heymans Institute of Pharmacology, Ghent University Hospital, De Pintelaan 185, B-9000 Gent, Belgium; 7 IBiTech-bioMMeda, Ghent University Hospital, De Pintelaan 185, B-9000 Gent, Belgium; 8 Pediatric Endocrinology, Department of Pediatrics and Turner Clinic, Ghent University Hospital, De Pintelaan 185, B-9000 Gent, Belgium

## Abstract

**Background:**

To study segmental structural and functional aortic properties in Turner syndrome (TS) patients. Aortic abnormalities contribute to increased morbidity and mortality of women with Turner syndrome. Cardiovascular magnetic resonance (CMR) allows segmental study of aortic elastic properties.

**Method:**

We performed Pulse Wave Velocity (PWV) and distensibility measurements using CMR of the thoracic and abdominal aorta in 55 TS-patients, aged 13-59y, and in a control population (*n* = 38;12-58y). We investigated the contribution of TS on aortic stiffness in our entire cohort, in bicuspid (BAV) versus tricuspid (TAV) aortic valve-morphology subgroups, and in the younger and older subgroups.

**Results:**

Differences in aortic properties were only seen at the most proximal aortic level. BAV Turner patients had significantly higher PWV, compared to TAV Turner (*p* = 0.014), who in turn had significantly higher PWV compared to controls (*p* = 0.010). BAV Turner patients had significantly larger ascending aortic (AA) luminal area and lower AA distensibility compared to both controls (all *p* < 0.01) and TAV Turner patients. TAV Turner had similar AA luminal areas and AA distensibility compared to Controls. Functional changes are present in younger and older Turner subjects, whereas ascending aortic dilation is prominent in older Turner patients. Clinically relevant dilatation (TAV and BAV) was associated with reduced distensibility.

**Conclusion:**

Aortic stiffening and dilation in TS affects the proximal aorta, and is more pronounced, although not exclusively, in BAV TS patients.

Functional abnormalities are present at an early age, suggesting an aortic wall disease inherent to the TS. Whether this increased stiffness at young age can predict later dilatation needs to be studied longitudinally.

**Electronic supplementary material:**

The online version of this article (doi:10.1186/s12968-017-0331-0) contains supplementary material, which is available to authorized users.

## Background

Turner syndrome (TS), occurring in approximately one in 2500 live born girls [1], is associated with left sided congenital cardiovascular defects. A bicuspid aortic valve (BAV) is found in up to one third of TS patients [2]. Aortic arch abnormalities such as a dysmorphic aorta or coarctation can be found in up to 50% of the patients [3–5]. Turner patients have increased risk of acquired aortic disease such as progressive dilation of the ascending aorta and dissection [2, 4, 6–11]. These aortic abnormalities contribute to the increased cardiovascular morbidity and mortality of women with Turner syndrome [4, 6, 12, 13]. Aortic dissection is a well-known fatal complication in TS patients and often occurs at a much younger age than in the general population. Detection of the patient at increased risk is difficult. The presence of a BAV, coarctation of a dilated aorta, as well as hypertension heralds higher risk, and warrants closer follow up [14]. But regardless of the presence of risk factors, follow up of aortic diameters has been inadequate at reliably predicting cardiovascular risk [15].

Besides excellent morphologic imaging, cardiovascular magnetic resonance (CMR) also allows assessment of segmental functional elastic properties of the aorta. Loss of aortic elasticity of the proximal aorta increases the left ventricle’s afterload and is related to increased cardiovascular risk [16]. In the older population as well as in several disease states, arterial stiffness has been shown to increase with age and to contribute to the pathogenesis of systolic hypertension and cardiovascular disease (end stage renal disease, hypertension, and coronary artery disease) [17–22].

Although numerous invasive and non-invasive descriptors of aortic elastic properties have been described, most outcome data center on aortic pulse wave velocity (PWV) [23] and distensibility [20, 22, 24, 25]. Previously we have shown that age-associated loss of elasticity is more pronounced in the thoracic aorta compared to the abdominal aorta in healthy controls [26]. We now hypothesized that Turner syndrome is associated with proximal aortic stiffening, and investigated the influence of age on this stiffness increase. To our knowledge, this study is the first to investigate segmental aortic properties in Turner patients by means of CMR.

## Methods

### Patient population

Fifty-five consecutive female Turner patients, aged between 13 and 59 years, referred for routine morphologic CMR of the aorta, were prospectively included. Pregnant or lactating women, patients with pacemakers or implantable cardioverter defibrillators (ICD’s), aneurysm clips, cochlear implants, neural stimulators, epileptic seizures, large tattoos, significant claustrophobia or morbid obesity that would not enable the subject to fit in the scanner, were excluded from the study. Patients who had had aortic valve replacement or arch stenting/surgery were excluded. The control group consisted of 38 apparently normal female subjects, aged between 12 and 58 year, recruited for a previous study on aortic PWV [26]. MR studies in all patients and controls were performed at the Ghent University Hospital, Belgium and reviewed by a single radiologist with 10 years of experience in cardiovascular CMR (DGHD). Body height and weight were measured prior to the MR examination. BMI and BSA, using the formula of weight divided by body height squared (kg/m^2^) and Mosteller [27] respectively, were calculated. We consulted the treating physicians and reviewed the patient medical records for documentation of estrogen replacement therapy (ERT). The study protocol was reviewed and approved by the Ghent University ethics committee. All patients and controls gave a written informed consent.

### MR scan protocol

All patients and controls were scanned on a 1.5 T magnet (Siemens, Erlangen). The scan protocol consisted of HASTE images of the thorax and abdomen (TR: 800 ms TE: 28 ms, FA: 160° ST: 6 mm). On four locations along the aorta, a retrospectively ECG-gated gradient-echo sequence with velocity encoding perpendicular to the aorta was applied to measure through-plane flow velocity (TR: 73 ms, TE: 4 ms, FA: 30°, ST: 6 mm, velocity sensitivity VENC: 150 cm/s, reconstructed phases: 40): ascending and descending aorta at the level of the pulmonary trunk were imaged in one series except in case of an extremely tortuous aorta, where two separate image planes were used; another series was placed at the level of the diaphragm, and a most distal series just above the aortic bifurcation [16]. A balanced fast field echo cine series with equal pixel matrix was acquired in exactly the same orientation (TR: 26–30 ms, TE: 1.2–1.5 ms, FA: 65–80, ST: 6 mm, number of images per cardiac interval: 40). During this breath hold, blood pressure was measured with a non invasive blood pressure monitor, using an adapted to size blood pressure cuff around the left upper arm (Tesla NIBP, Mammendorf, Germany). Baseline blood pressure was the average of all measurements that were performed during transverse scans.

In addition a standard keyhole high temporal, high spatial resolution MRA sequence of the entire aorta (Isovolumetric (1.3 mm) matrix 240x320 pixels, FOV: 420x320mm, TR: 2.6 ms, TE: 0.9 ms, symmetrically shared 33% peripheral K-space data for 20% central data (TWIST, time-resolved angiography with stochastic trajectories) was performed in patients. Aortic arch morphology was classified as normal arch (Romanesque, no stenosis and no arteria subclavia lusoria) or elongated transverse arch, tortuous arch, crenel shaped arch or gothic arch [4].

### Postprocessing

Postprocessing was performed off-line by a single radiologist (DGHD). It consisted first of manually drawing a centerline in the aortic lumen on a parasagittal angiographic planar reformatted image (so called candy cane view of the aorta). Along this centerline, the distance (Δx) was measured between the positions where the Phase Contrast images of the ascending aorta, the aorta at diaphragmatic level, and just above the aortic bifurcation were made (Additional file 1: Figure S1). On these phase contrast images the aortic lumen was segmented in an automated fashion [28]. Using in-house developed MATLAB code, the last 5 data points of each flow-time curve were cut and pasted in front of the time series, in order to translate the curve in time, ensuring that the onset of each curve was positioned after the start of the revised time series (Additional file 2: Figure S2). The graphs were subsequently processed in a custom-made PWV analysis tool as described by Grotenhuis et al. [29]: the onset of the systolic wave front was automatically determined from the resulting flow graph by the intersection point of the constant horizontal diastolic flow and the upslope of the systolic wave front, the latter of which was modeled by linear regression along the upslope from the flow values between 20% and 80% of the total range (Additional file 3: Figure S3). PWV was calculated as the ratio of distance Δx per time Δt, where Δx is the length of an aortic segment (thoracic, abdominal or entire aorta, see Additional file 1: Figure S1) measured on the MR image data along the centerline, and Δt is the time duration needed for the pulse wave velocity to travel that length through the aorta.

Maximum and minimum transverse aortic luminal areas were manually located and drawn on the cine series. Distensibility was calculated as:

$$ \mathrm{D} = \left(\mathrm{Amax}\hbox{-} \mathrm{Amin}\right)/\left(\mathrm{Amin}\ *\ \left(\mathrm{Pmax}\hbox{-} \mathrm{Pmin}\right)\right) $$

where Amax and Amin are maximum and minimum area, Pmax is peak systolic blood pressure and Pmin is minimal (diastolic) blood pressure; it is expressed as 1/1000*mmHg. These blood pressures were taken from left arm sphygmomanometric measurement during the actual distensibility scan.

### Statistical analysis

Statistical exploration was performed in Wizard v1.8.9 (OSX El Capitan v10.11.4).

Statistical analysis was performed in SPSS for Windows (version 22; SPSS, Chicago, Illinois, USA). Normality of distribution was explored with histograms and Q-Q plots. Baseline data are expressed as mean (standard deviation; SD) and compared with an independent T-test if normally distributed. PWV values were expressed as median (interquartile range) and compared using the Kruskal Wallis test. For the multivariate analyses General Linear Modeling was used adjusting for age, height, weight, heart rate and systolic blood pressure as covariates. The dependent variables in these models were ln-transformed PWV, distensibility and area values because of some skewness towards higher values. The level of significance was set at *p* < 0.05.

## Results

### Population characteristics

Clinical characteristics and cardiac parameters of both the control and Turner population are listed in Table 1. The Turner population was significantly shorter, and had a higher BMI compared to controls. Haemodynamically, TS patients had a similar mean systolic blood pressure compared to controls, but presented with a higher mean heart rate and diastolic blood pressure. Mean left ventricular volume including stroke volume was smaller in TS patients, even after normalization for Body Surface Area (BSA). There was no significant difference in mean left ventricular ejection fraction, nor in left ventricular mass normalized for body surface area. As expected, TS patients, being smaller, had significantly shorter descending thoracic aorta and abdominal aortic lengths. In contrast, their aortic arch length was similar to the controls’. While all controls had tricuspid aortic valves, 16 Turner patients had a bicuspid aortic valve (BAV).

**Table 1:** Population characteristics

	Control	Turner	*p* value
	(*n* = 38)	(*n* = 55)	
Age (y)	34 (14)	29 (11)	0.060
Length (cm)	166 (8)	154 (7)	**<0.001**
Weight (kg)	61 (11)	57 (11)	0.159
BMI (kg/m^2^)	21.8 (3.1)	24.3 (4.3)	**0.003**
BSA (Mosteller)	1.67 (.17)	1.56 (0.17)	**0.003**
Systolic BP (mmHg)	118 (14)	121 (16)	0.259
Diastolic BP (mmHg)	70 (9)	81 (12)	**<0.001**
Heart Rate (bpm)	69 (10)	79 (13)	**<0.001**
Bicuspid aortic valve (%)	0%	16%	
Aortic arch length (mm)	97 (13)	102 (20)	0.158
Descending aorta (mm)	108 (15)	100 (13)	**0.008**
Abdominal aorta (mm)	170 (21)	158 (20)	**0.007**
Thoracic aortic PWV (m/s) ^a^	3.7 (1.7)	4.4 (1.7)	0.127
Abdominal aortic PWV (m/s) ^a^	4.7 (2.5)	4.8 (1.6)	0.829
Overall aortic PWV (m/s) ^a^	4.1 (1.6)	4.4 (1.2)	0.23

Values are Mean (standard deviation), comparison using unpaired t-test. All variables normally distributed, except PWV values (^a^) which are expressed as median (interquartile range), comparison using the Kruskal-Wallis test. Bold indicates <0.05

### Comparison of aortic measurements in Turner patients and in controls

The multivariate determinants of aortic wall properties were assessed in a Generalized Linear Model (GLM) including the presence of Turner syndrome and adjusting for possible confounders: age, height, weight, heart rate and systolic blood pressure. Age was included as a covariate to take into account the potential effect of the non-significant age difference between Turners and controls.

**Fig. 1:**
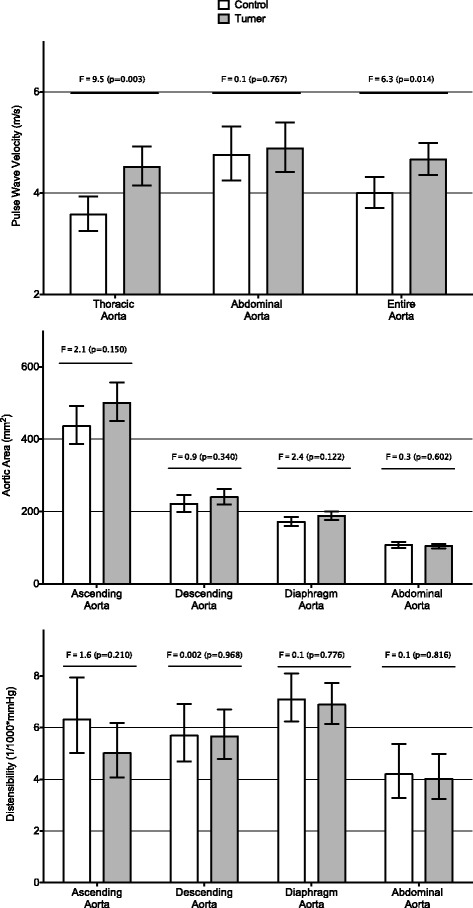
PWV in thoracic, abdominal and entire aorta, aortic area and distensibility of ascending and descending aorta as well as at the level of the diaphragm and distal abdominal aorta. Comparison of controls (*white*) vs Turner Syndrome (*shaded*). Data (estimated marginal means and 95% confidence interval) and *p*-values were derived from a multivariate GLM including age, height, weight, heart rate and blood pressure as confounders

The effect of the presence or absence of Turner syndrome is shown in Fig. 1 where estimated marginal means were plotted for thoracic, abdominal and total aortic PWV, area and distensibility in Turner patients versus controls. As expected in all models, age was the strongest determinant. Turner patients had significantly higher thoracic aortic PWV (TA-PWV) compared to controls. Abdominal PWV was similar, the higher overall aortic PWV in Turner subjects being driven by the higher TA-PWV. No statistically significant differences in aortic area and distensibility were seen between Turner patients and controls.

### The impact of aortic valve morphology on aortic parameters

We then analysed the impact of aortic valve morphology. Table 2 shows the baseline characteristics of the BAV Turner patients, Tricuspid Aortic Valve (TAV) Turner patients, and controls. There were no significant differences between baseline population characteristics of TAV and BAV Turner patients.

**Table 2:** Subgroup characteristics and comparison

	TAV Control	TAV Turner	BAV Turner	Control-TAV Turner	Control-BAV Turner	TAV- BAV Turner
	(*n* = 38)	(*n* = 39)	(*n* = 16)	*p*	*p*	*p*
Age (y)	34 (14)	30 (12)	25 (9)	0.241	**0.008**	0.128
Length (cm)	166 (8)	154 (8)	152 (6)	**0.000**	**0.000**	0.466
Weight (kg)	61 (11)	58 (12)	55 (8)	0.329	0.099	0.458
Heart Rate (bpm)	69 (10)	78 (14)	83 (8)	**0.002**	**0.000**	0.120
BMI (kg/m^2^)	21.8 (3.1)	24.4 (4.8)	23.9 (2.8)	**0.006**	**0.032**	0.589
BSA (mosteller)	1.67 (0.17)	1.6 (0.2)	1.5 (0.1)	**0.016**	**0.007**	0.454
Systolic BP (mmHg)	118 (14)	123 (16)	118 (14)	0.151	0.982	0.316
Diastolic BP (mmHg)	70 (9)	82 (14)	80 (9)	**0.000**	**0.002**	0.528
Aortic arch length (mm)	97 (13)	99 (15)	110 (27)	0.542	**0.021**	0.055
Descending aorta (mm)	108 (15)	102 (13)	96 (12)	0.050	**0.009**	0.159
Abdominal aorta (mm)	170 (21)	156 (21)	163 (16)	**0.005**	0.235	0.245

Descriptive parameters per subgroup, with *p*-value (t-test; bold indicates <0.05) for each comparison of two subgroups

**Fig. 2:**
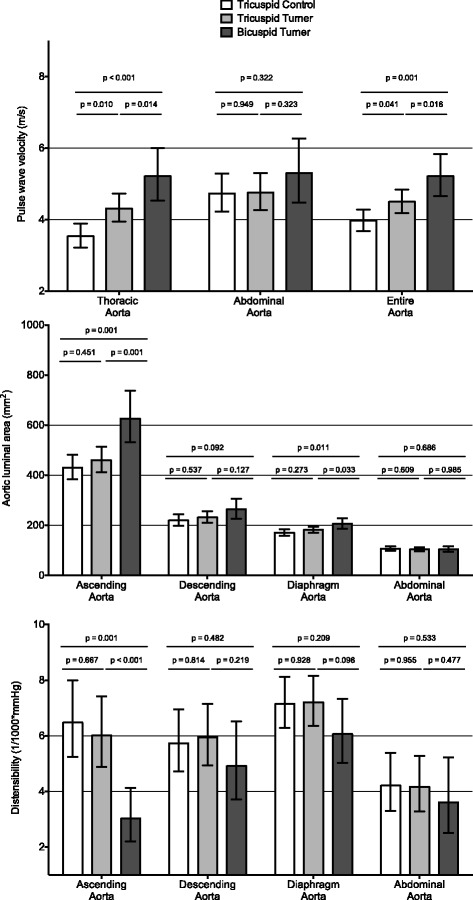
PWV in thoracic, abdominal and entire aorta, aortic area and distensibility of ascending and descending aorta as well as at the level of the diaphragm and distal abdominal aorta. Comparison of TAV controls, TAV Turners and BAV Turners. Data (estimated marginal means and 95% confidence interval) and *p*-values were derived from a multivariate GLM including age, height, weight, heart rate and blood pressure as confounders

To assess the effect of aortic valve on the aortic properties, we performed a GLM analysis comparing controls, TAV Turner and BAV Turner, again adjusting for age, height, weight, heart rate and systolic blood pressure. The results are plotted in Fig. 2, showing the multivariate adjusted estimated marginal means (and 95% confidence interval) of the studied aortic parameters. Overall, differences in aortic properties were seen at the most proximal aortic level: BAV Turner patients had significantly higher PWV, compared to TAV Turner (*p* = 0.014), who in turn had significantly higher PWV compared to controls (*p* = 0.010). BAV Turner patients had significantly larger AA luminal area and lower AA distensibility compared to both controls (all *p* < 0.01) and TAV Turner patients. TAV Turner had similar ascending aortic luminal areas and ascending aortic distensibility compared to Controls.

No consistent differences were seen across groups in more distal aortic characteristics. Exceptions were marginal (but significant) differences in diaphragmatic area (larger in BAV Turner patients), and differences in overall aortic PWV (Turner higher than controls), again driven by the differences in thoracic PWV, as abdominal PWV values were similar.

Adding left ventricular stroke volume, which was significantly smaller in Turners compared to controls, to these models did not substantially change the results (data not shown).

### The impact of age on aortic parameters

We plotted F and *p* values of the used GLM model within the Turner group and the control group separately in Additional file 4: Table S1. Age is the main determinant of PWV, aortic luminal area and distensibility at all levels in both groups, only surpassed slightly by systolic blood pressure for descending and distal abdominal distensibility in Turner patients (and weight for distal abdominal aortic area in the control group).

Using the same model separately in the TAV Control group, in the TAV Turner patient group and in the BAV Turner patient group (F and *p* values in Additional file 5: Table S2), the independent association between aortic properties and age remain largely unchanged in both TAV groups. Strikingly, in BAV Turners almost all effects of age, blood pressure, height and weight were lost.

**Fig. 3:**
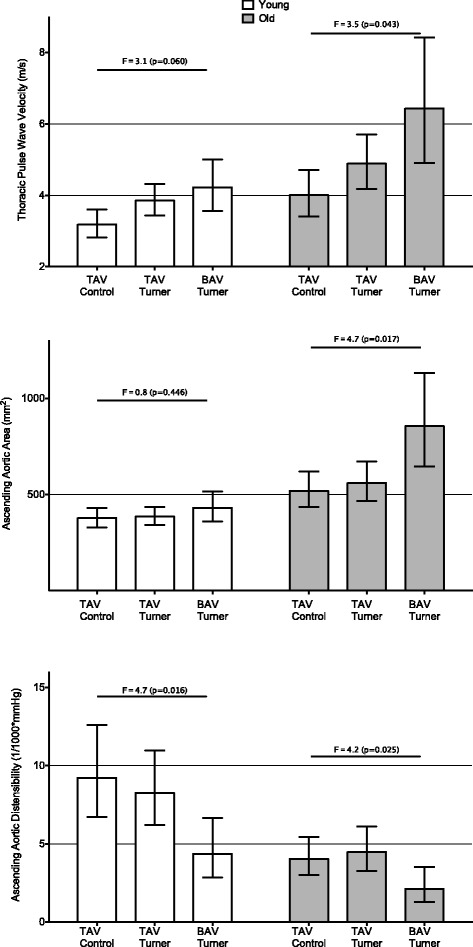
Thoracic PWV, ascending aortic area and distensibility. Comparison younger half (*white*) vs older half (*shaded*) in TAV control group, TAV Turner group and BAV Turner group. Data (estimated marginal means and 95% confidence interval) and *p*-values were derived from a multivariate GLM including age, height, weight, heart rate and blood pressure as confounders

To further study whether the impact of Turner Syndrome on aortic properties is different in younger versus older subjects, we divided all three subgroups into two halves, above and below their respective median age. Age cut-offs were 27 years for TAV Turner subjects; 23.5 years for BAV Turner subjects; 32 years for Controls. A similar GLM model adjusting for age, height, weight, heart rate and systolic blood pressure was used and the results of proximal aortic properties are shown in Fig. 3. Overall, the functional changes (borderline increase in thoracic PWV and significant loss of proximal aortic distensibility) are present in both younger and older Turner subjects, whereas structural remodeling (enlargement of ascending aortic) clearly is only prominent in older BAV Turners.

**Fig. 4:**
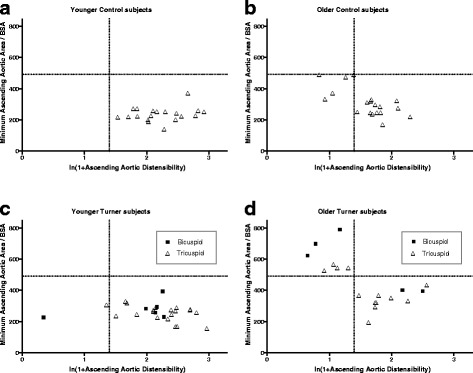
Scatterplots of ascending aortic area (BSA-normalised) and distensibility (incremented and ln-transformed) in young (**a**) and old (**b**) Controls and in young (**c**) and old (**d**) Turner patients, illustrating an inverse relation in the older half of both populations, irrespective of aortic valve morphology (triangle = tricuspid valve; square = bicuspid valve). The reference line at a normalised ascending aortic area of 491 mm^2^/m^2^ corresponds to the cut-off aortic diameter of 2.5 cm/m^2^

Finally, we plotted the ascending aortic area in relation to the ascending aortic distensibility for young and old groups of controls, TAV and BAV Turner patients (Fig. 4). This graph illustrates the inverse correlation between ascending aortic area (BSA-normalised) and its distensibility (incremented and ln-transformed), or, larger aortas are stiffer in older controls and Turner patients (*p* < 0.001), regardless of aortic morphology. There is no such correlation in the younger subjects (either controls or Turner patients).

### Additional results

We found ERT in at least 43 of 55 Turner patients (78%). Four patients previously received ERT, but it was stopped or temporarily interrupted in the year of the MR scan. Retrospectively, we could not rule out that the remaining 8 patients ever had been on ERT (5 Turner patients probably not on ERT, and unknown in 3).

Aortic arch morphology was normal (Romanesque, no stenosis, no lusoric subclavian artery) in all 38 control subjects, compared to only 20 out of 55 Turner patients. Thirty-five Turner patients had aortic arch abnormalities: 21 elongated transverse arches, 5 tortuous arches, 4 crenel shaped arches, 2 gothic arches, 6 subjects had an aortic arch stenosis (5/6 were moderate stenosis), and 8 subjects had an arteria subclavia lusoria. Adding aortic arch abnormalities to the GLM models did not impact the models for PWV, but for AA distensibility and for AA area, the aortic arch abnormalities competed with the diagnosis of Turner Syndrome and/or presence of a bicuspid aortic valve (i.e. partial co-linearity, especially with BAV presence).

## Discussion

In this study, we evaluated PWV, luminal area and distensibility of both the thoracic and abdominal aortic segments in 55 Turner patients compared to controls. The main findings of our study are that (i) only the proximal aorta is significantly stiffer (PWV) in Turner patients compared to controls, independently of body height, (ii) the ascending aorta is significantly less distensible and also more enlarged in Turner patients with a bicuspid aortic valve compared to Turner patients with a tricuspid aortic valve, and (iii) the functional proximal aortic changes were largely similar when comparing younger and older TS patients, but aortic dilatation (more pronounced in BAV Turner patients) seems acquired/progressive, although this interpretation should be seen as purely hypothesis-generating due to the cross-sectional nature of our data. Those subjects with clinically relevant dilatation (>2,5 cm/m^2^) had a markedly reduced distensiblity. The scatterplot between AA area/BSA and distensibility shows a distinct (almost bimodal) distribution with dilated and stiff AA clearly clustering compared to non-dilated and less stiff AA.

Our data supports the previously reported dilation of the proximal segment of the aorta in Turner syndrome [28, 30–32]. Matura et al. found that 24% of TS patients had dilatation of the ascending aorta defined as exceeding the 95th percentile of BSA-adjusted aortic diameter for age-matched control women, and they proposed that this group required close cardiological surveillance. Within this cohort of dilated ascending aorta in TS, they identified a population of TS at highest risk for dissection with a cut-off of BSA-adjusted aortic diameter >2.5 cm/m^2^ or an absolute diameter >3.5 cm [28]. Extrapolating from these cut-offs we calculated that a BSA-adjusted aortic diameter >2.5 cm/m^2^ would equate a BSA-adjusted area of >4,91 cm^2^/m^2^. This cut-off was exceeded in our cohort by 11.4% of TAV Turner patients, 26,7% of BAV Turner patients, and none of the controls (Chi-square test; *p* = 0.008). It is noteworthy that those Turner subjects with dilated ascending aortas were characterized by a clearly lower ascending aortic distensibility (see Fig. 4). In 2011, Mortensen reported that the ascending aorta in TS is diseased and dilated, and that the proximal aortic diameter growth rate was 0.1–0.4 mm/year; presence of a BAV was associated with a significantly increased growth rate [30].

Whilst it has been reported –using various methodologies- that Turner patients have a stiffer aorta compared to controls [33–35], this is the first study using CMR PWV to assess the functional properties of the aorta at various aortic levels. Indeed, we confirm the previous findings that Turner subjects have stiffer arteries, but extend this by documenting a preferential involvement of the proximal aorta, especially in patients with a bicuspid aortic valve. In addition, BAV Turner patients had a (borderline) non-significantly longer aortic arch compared to TAV Turner patients. An elongated mid arch segment or more serious deformations of the entire arch [36] have been reported in these patients. It could be that a more precise way of quantifying aortic arch length and deformation allows for better differentiation of abnormality, and the relative impact on PWV of both aortic arch morphology and aortic wall stiffness in Turner syndrome.

In the younger half of our study subjects we found a significantly lower ascending aortic distensibility and borderline non-significant higher PWV of the thoracic aorta in BAV Turner patients, but no significant difference in ascending aortic transverse area. In the older half of our study population, we found significantly increased thoracic aortic PWV and ascending aortic area, as well as significantly decreased ascending aortic distensibility. However, correlation of ascending aortic area and distensibility shows that the presence of a bicuspid valve cannot on its own predict dilatation of the ascending aorta. As aortic wall stiffness seems increased at early age, before dilatation is detectable, we put forward the idea to study the possible prediction of proximal aortic dilatation from early distensibility measurements in further longitudinal studies.

The association of Turner syndrome with aortic wall disease presenting from young age, as we found in our data, is consistent with reports that increased risk of acute aortic dissection is present from as early as the second decade of life [1, 12, 13], and that dissection may occur as early as the first decade of life [37]. Gravholt et al. have estimated the incidence of dissection of 36 per 100,000 Turner’s syndrome years, compared with an incidence of 6 per 100,000 in the general population [38]. Meszaros found an incidence of dissection of 2.9/100,000 patient years in a population study of 100,000 patients during 27 years in the hospitals of three adjacent small towns in western Hungary [39]. Turner patients are at risk of aortic dissection at a significantly younger age (31–35 years) [38, 40] than the normal female population (68 years) [41]. An et al. have reported recently that the aortic arch, measured by ultrasound, was not dilated but less elastic in 25 adolescent Turner patients compared to controls [42].

Our data support the closer follow up of those TS patients with a bicuspid aortic valve [7].

Which TAV Turner patients should be followed up equally frequently, all or only a subset with stiffer aorta at young age remains to be studied.

Whether TAV Turner patients can be followed up safely with less frequent re-examinations needs to be further studied. Current guidelines recommmend ‘full evaluation with thoracic CMR at an age when it can be performed without sedation’ in young Turner patients [43]. A practical approach could be to initially use ultrasound, and refer to CMR if the aorta cannot adequately be measured, and perform CMR at the age of 10 years; further follow up is currently guided by BSA normalized aortic diameters and the presence of risk factors [44].

### Study limitations

A limitation of our study is the small number of patients, especially when groups were divided in subgroups: the total number of bicuspid Turner patients was only 16. In addition the effect of estrogen replacement therapy (ERT) and previous growth hormone therapy was not studied. We did review the patient files for history of ERT, but with the vast majority of Turner patients on ERT, we were not able to divide into ERT/No ERT groups of sufficient size. However, the effect of ERT on aortic diameter was not statistically significant (*p* = 0.08) in a prospective 5 year follow up study (*n* = 78) [15]. In a smaller study the same group has investigated the effect of 6 months of ERT on ambulatory arterial stiffness index (AASI) derived from 24-hour ambulatory blood pressure: although Turner patients were found to have higher AASI when compared to controls, AASI was unchanged by ERT [45].

The age difference between control population and the 5 years younger Turner population was not significant, but once subdivided in valve morphology groups, the small BAV Turner patients were significantly younger than the TAV Turner patients. The older/younger subdivision of our control and Turner patients at the respective median line was chosen to preserve equal number of subjects in both young and old groups. Subdivision at one fixed age for all groups would hinder proper statistical analysis, as this would lead to comparing a large group of young Turner patients, with a small group of young controls.

Blood pressure measurement was performed during the actual distensibility scan, but we were limited to a left arm sphygmomanometric measurement. This may not be representative for the blood pressure in the ascending aorta proximal to a dysmorphic arch.

The postprocessing of PWV is another limitation. Detection of the foot of the curve is difficult in such a fast physiologic process, when performed with the relatively poor temporal resolution of MR. However this limitation applies equally to all studied groups in this study. Our data did not allow analyzing smaller aortic segments. Other studies have focused on increasing temporal resolution by acquiring in-plane velocity encoded images [46, 47]. At the time our data acquisition started, transverse images and detection of the foot-of-the-curve time shift was the major proposed method, a transit-time method focussing at the early systolic part of the curve in an attempt to avoid the effect of wave reflection, which, especially in a stiffer aorta, is important. It was used as early as 1993 by Mohiaddin et al. [48] and more recently, by means of computer phantoms, Dorniak et al. calculated the minimal required temporal resolution of 30 ms (35 image frames at a heartrate of 60 bpm) for accurate and precise quantitative flow data for CMR-PWV over the range 2–20 m/s in the thoracic aorta [49]. Newer methods involve complex wavelet cross spectrum analysis [50], which equally have the advantage of being robust at lower temporal resolution.

Our transverse aortic area measurements were not corrected for longitudinal strain, which in the ascending aorta reduces systolic dilation and thus leads to underestimation of distensibility [51]. However, ascending and descending aortic distensibilities in our study are comparable. To minimize through-plane motion error, the ascending aortic cine image was positioned carefully out of range of the motion of the sinotubular junction. The reported perpendicular aortic areas in our work are end-diastolic. As for comparison with other PWV values in literature, it is important to note that the most critical aspect of PWV postprocessing is the lack of standardization and all-in-one software solutions.

Our data is not longitudinal. We can therefore not make assumptions regarding the evolution of a stiffer proximal aorta at young age towards dilatation at older age. We do however think this should be studied further.

## Conclusion

In conclusion, Turner patients exhibit a predominantly proximal aortic stiffening and dilatation, especially -but not exclusively- if their aortic valve is bicuspid. Aortic stiffening in Turner syndrome is already present at an early age, whereas ascending aortic dilatation is not. We put forward the hypothesis that proximal aortic stiffness at early age may predict aortic dilatation in both bicuspid and tricuspid aortic valve Turner patients. This hypothesis should be studied longitudinally.

## Novelty and significance

### What is new?

We have studied *segmental thoracic versus abdominal* aortic Pulse Wave Velocity acquired with CMR in Turner patients.

### What is relevant?

In Turner patients, structural (dilatation) and functional aortic wall changes (stiffening and loss of distensibility) are restricted to the proximal aorta, even more so –but not exclusively- in the presence of a bicuspid aortic valve.

Increased thoracic aortic wall stiffness is already present at a young age whereas the aortic dilatation was only seen in our older Turner patients, specifically in those with the most pronounced stiffening.

If proximal aortic stiffness measurements could be used to predict dilatation -and by extension, risk of dissection- at older age, the follow-up criteria and timing of both bicuspid and tricuspid aortic valve Turner patients could be redefined.

### Summary

Turner patients exhibit a predominantly proximal aortic stiffening and dilatation, especially -but not exclusively- if their aortic valve is bicuspid. Aortic stiffening in Turner syndrome is already present at an early age, whereas ascending aortic dilatation is not. We put forward the hypothesis that proximal aortic stiffness at early age may predict aortic dilatation in both bicuspid and tricuspid aortic valve Turner patients. This hypothesis should be studied longitudinally.

## Additional files

Additional file 1: Figure S1. Distance measurement along the aortic path. Aortic segment and abdominal segment length are measured. (PDF 166 kb)
Additional file 2: Figure S2. Transposition of 5 last curve data points to the left so as to view and analyze the foot of the curve. (PDF 69 kb)
Additional file 3: Figure S3. Determination of time interval between velocity curves based on each curve’s intersection point of the constant horizontal diastolic flow and the upslope of the systolic wave front. (PDF 69.5 kb)
Additional file 4: Table S1. Supplemental **Table S1** A and B. F and *p* values of PWV, aortic area and aortic distensibility in amultivariate GLM including age, height, weight, heart rate and blood pressure as confounders, performedseperately within each of both groups: Turner patients (S1A) and controls (S1B). (XLSX 17 kb)
Additional file 5: Table S2. Supplemental **Table S2** A, B and C. F and *p* values of PWV, aortic area and aortic distensibility in a multivariate GLM including age, height, weight, heart rate and blood pressure as confounders, performed seperately within each of three groups: TAV controls (S2A), TAV Turner patients (S2B) and BAV Turner patients (S2C). (XLSX 17 kb)

## Abbreviations

AA: Ascending aorta
AASI: Ambulatory arterial stiffness index
BAV: Bicuspid aortic valve
BMI: Body mass index
BP: Blood pressure
BSA: Body surface area
CMR: Cardiovascular magnetic resonance
EDLV: End diastolic left ventricular volume
ESLV: End systolic left ventricular volume
ETA: Elongation of the transverse arch
FA: Flip angle
FOV: Field of view
GLM: Generalized linear model
ICD: Implanted cardioverter defibrillator
LV: Left ventricle/ventricular
LVEF: Left ventricular ejection fraction
LVSV: Left ventricular stroke volume
MR: Magnetic resonance
MRA: Magnetic resonance angiography
PWV: Pulse wave velocity
SD: Standard deviation
ST: Slice thickness
TA: Thoracic aorta
TAV: Tricuspid aortic valve
TE: Echo time
TR: Repetition time
TS: Turner syndrome
TWIST: Time-resolved angiography with stochastic trajectories
VENC: Velocity ENCoding
