# Supplementary figures and images for: Proximal aortic stiffening in Turner patients may be present before dilation can be detected: a segmental functional MRI study
Source: J Cardiovasc Magn Reson. 2017 Feb 13;19:27. doi: 10.1186/s12968-017-0331-0 (PMC5320803; doi:10.1186/s12968-017-0331-0)

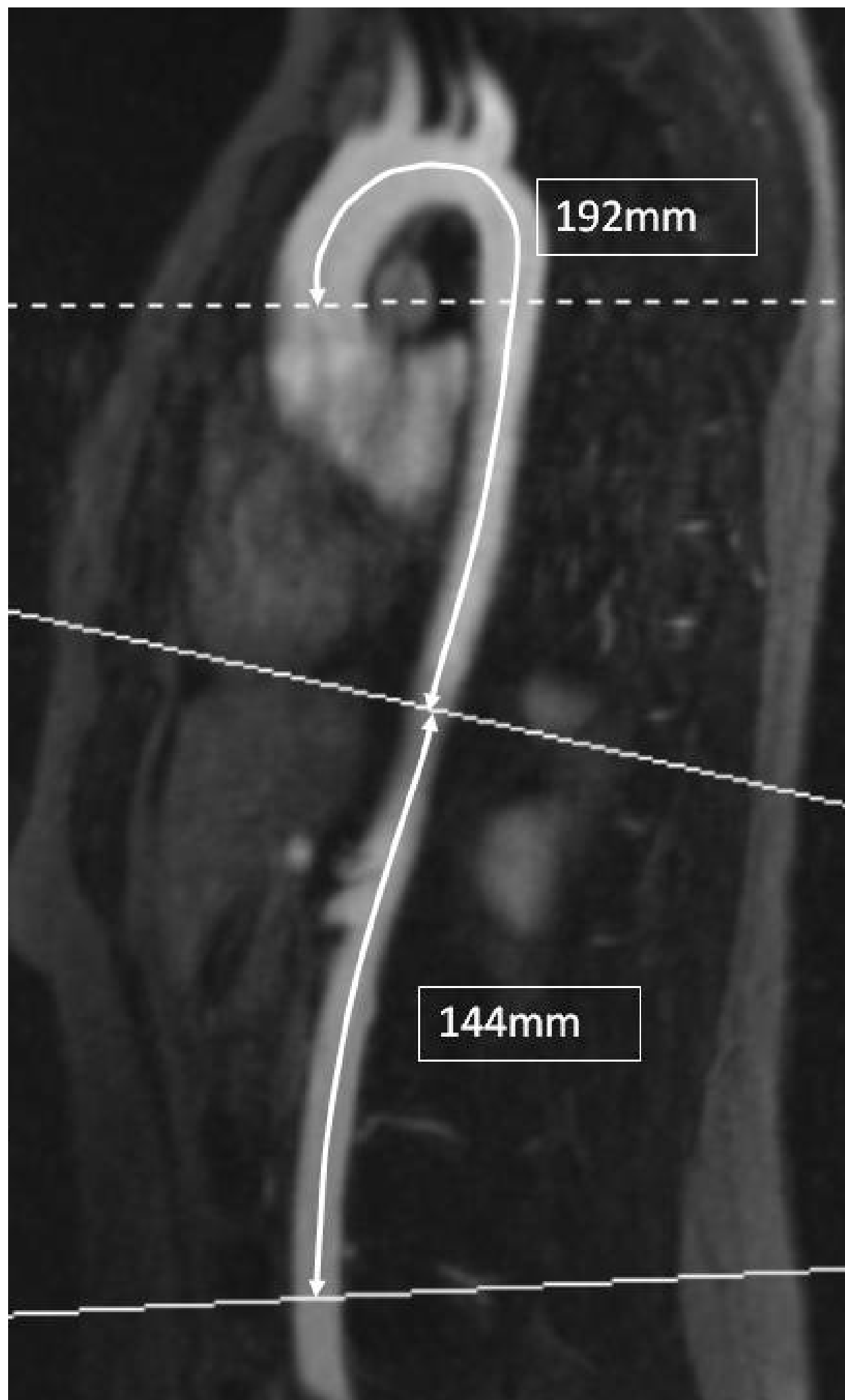

Supplement: Additional file 1: Figure S1. — Distance measurement along the aortic path. Aortic segment and abdominal segment length are measured. (PDF 166 kb) [file 12968_2017_331_MOESM1_ESM.pdf]

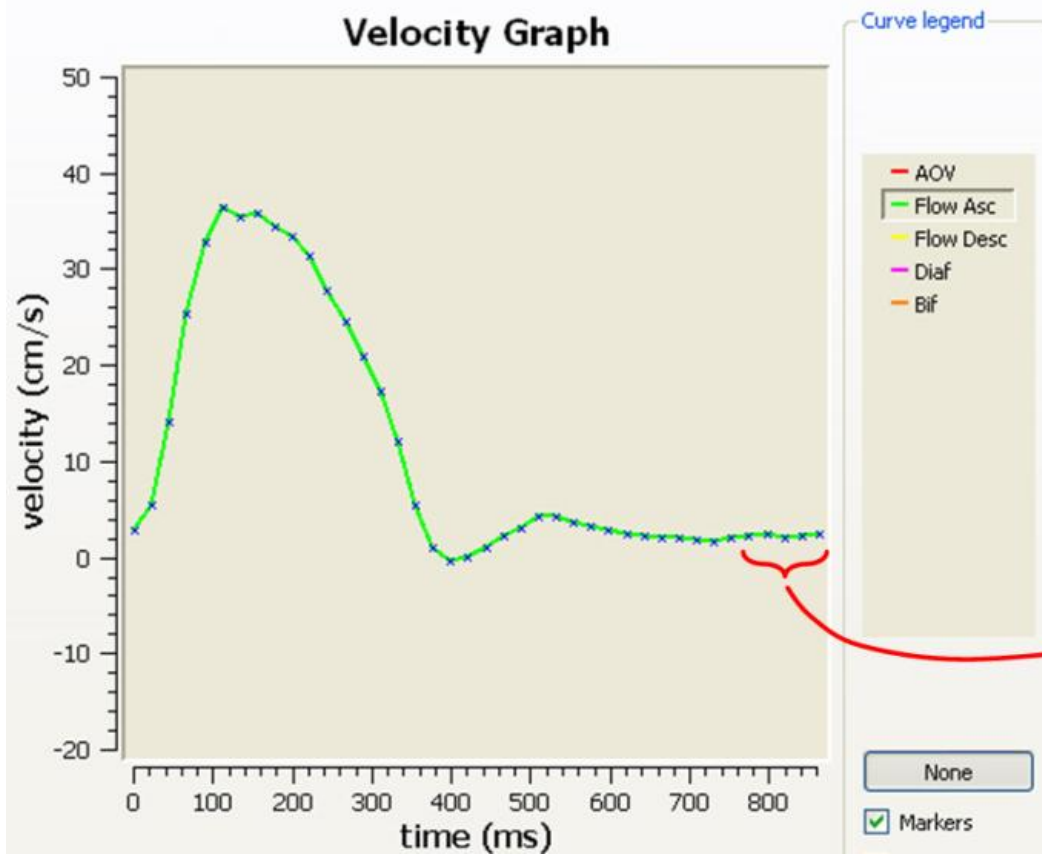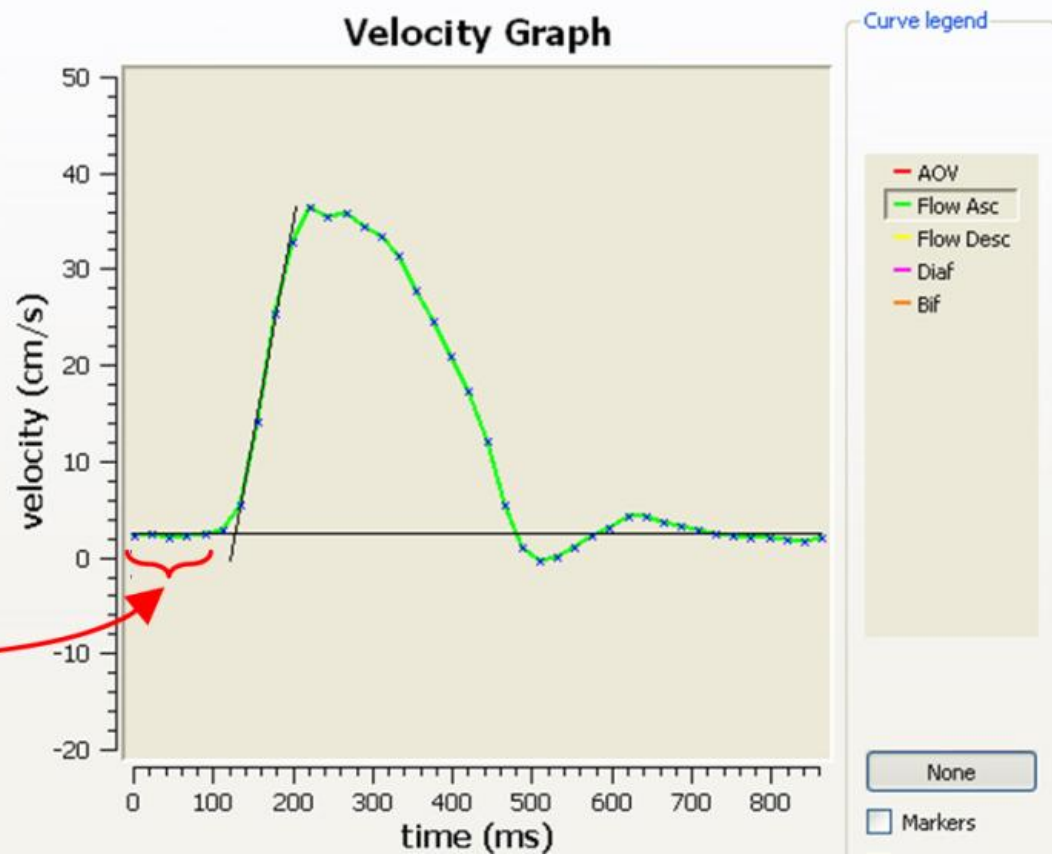

Supplement: Additional file 2: Figure S2. — Transposition of 5 last curve data points to the left so as to view and analyze the foot of the curve. (PDF 69 kb) [file 12968_2017_331_MOESM2_ESM.pdf]

# Velocity Graph

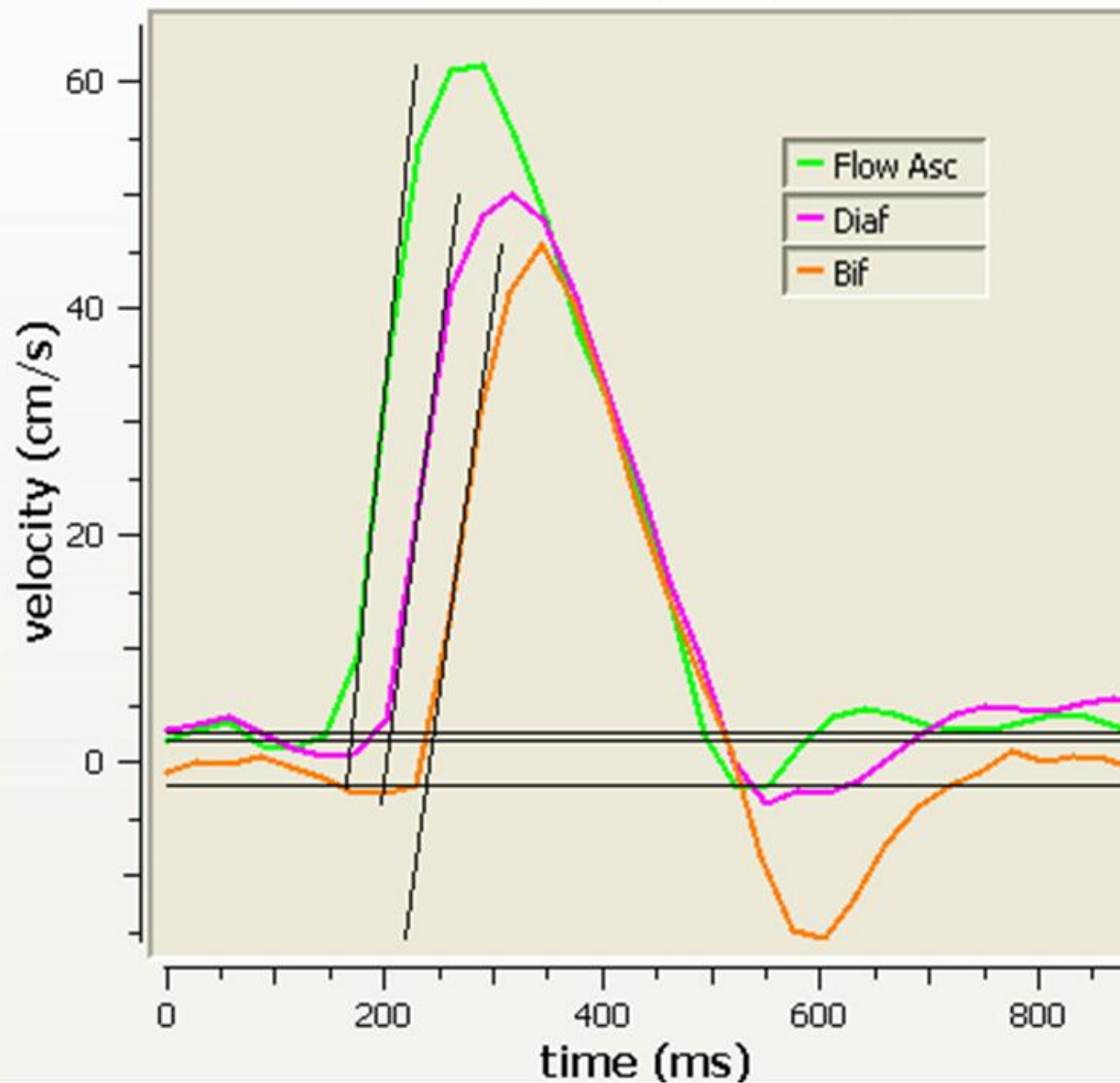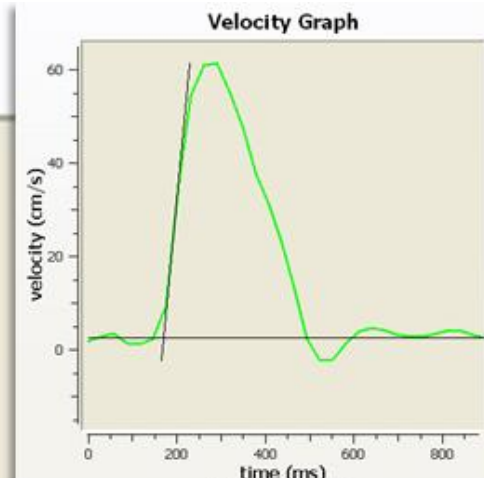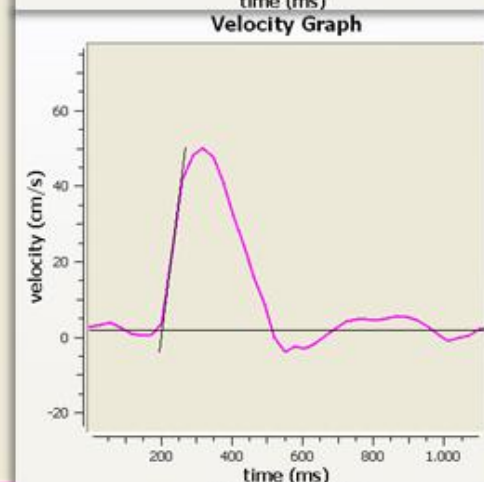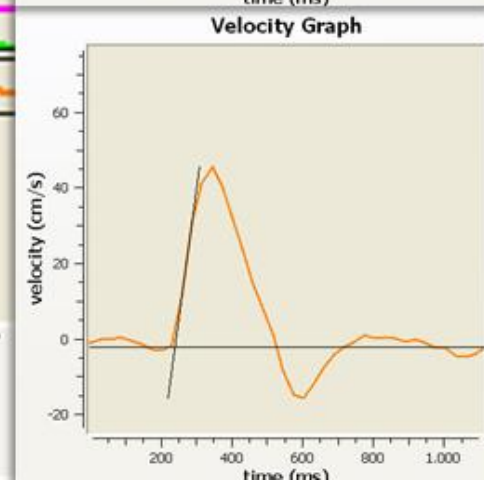

Supplement: Additional file 3: Figure S3. — Determination of time interval between velocity curves based on each curve’s intersection point of the constant horizontal diastolic flow and the upslope of the systolic wave front. (PDF 69.5 kb) [file 12968_2017_331_MOESM3_ESM.pdf]
